# Assessing the efficacy and safety of pentoxifylline in preventing chemotherapy-induced peripheral neuropathy and mucositis in breast cancer patients

**DOI:** 10.3389/fphar.2025.1678161

**Published:** 2025-10-14

**Authors:** Samar A. Dewidar, Noha O. Mansour, Omar Hamdy, Ahmed Eltantawy, Moetaza M. Soliman, Mohamed E. E. Shams

**Affiliations:** ^1^ Clinical Pharmacy and Pharmacy Practice Department, Faculty of Pharmacy, Mansoura University, Mansoura, Egypt; ^2^ Surgical Oncology Department, Oncology Center, Mansoura University, Mansoura, Egypt; ^3^ Medical Oncology Unit, Oncology Center, Mansoura University, Mansoura, Egypt

**Keywords:** breast cancer, neoadjuvant chemotherapy, pentoxifylline, neuropathy, mucositis

## Abstract

**Background:**

Pentoxifylline (PTX) has demonstrated potential in alleviating several adverse effects induced by chemotherapy in preclinical and limited clinical investigations. Nonetheless, its efficacy in mitigating the overall toxicity associated with doxorubicin, cyclophosphamide, and taxane (AC/T) regimen in breast cancer patients remains uncertain.

**Methodology:**

This study is an open labelled clinical trial in which the participants were randomly assigned to receive either PTX (400 mg three times daily) with the standard chemotherapy regimen or standard chemotherapy alone. The outcomes of the present study include the occurrence of grade 2 or higher peripheral sensory neuropathy and mucositis evaluated according to common terminology criteria for adverse events (CTCAE v5.0).

**Results:**

A total of 106 patients completed the study (PTX: 52; control: 54). The incidence of grade 2 or higher peripheral neuropathy was higher during the taxane regimen and was notably reduced in the PTX group relative to controls (75% vs. 90.7%, *P =* 0.03), accompanied by an extended duration until grade 2 or higher neuropathy onset in the PTX arm (log-rank *P <* 0.0001). PTX significantly decreased the occurrence of grade 2 or higher mucositis during both the AC and taxane phases (*P =* 0.01 and *P =* 0.04, respectively) without causing notable differences in hematological toxicities or affecting cardiac, renal, or hepatic functions.

**Conclusion:**

The co-administration of PTX with AC/T chemotherapy in breast cancer patients significantly decreases the incidence and postpones the onset of grade 2 or higher peripheral neuropathy and the incidence of mucositis without causing additional risks for the patients.

**Clinical Trial Registration:**

The study was registered at https://clinicaltrials.gov/study/NCT06186700 (NCT06186700).

## Graphical Abstract

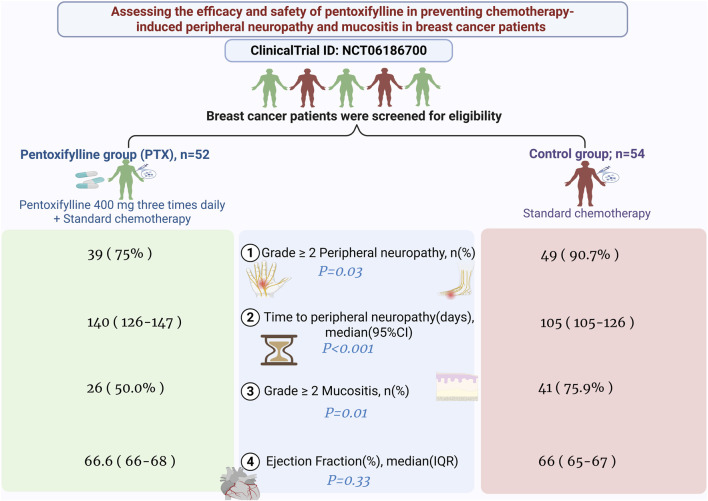

## 1 Introduction

Breast cancer represents an important health concern worldwide. In 2022, approximately 2.3 million new cases were diagnosed worldwide, with 670,000 related deaths (Kim et al., 2025). Chemotherapy remains a cornerstone of comprehensive breast cancer management, especially regimens including doxorubicin with cyclophosphamide followed by taxane (AC/T), which is the main treatment protocol for early and locally advanced stages of breast cancer (Gradishar et al., 2024). Nevertheless, despite its efficacy, this protocol has several distressing toxicities that may negatively affect patients’ quality of life.

Paclitaxel-induced peripheral neuropathy (PIPN) is a well-documented adverse effect, affecting up to 97% of patients receiving paclitaxel-containing regimens (Saleh et al., 2024; Zajączkowska et al., 2019). Similarly, doxorubicin is known for its cardiotoxicity and myelosuppression, while cyclophosphamide can cause hematological toxicity and immunosuppression (Chatterjee et al., 2010; Emadi et al., 2009). Mucositis is another common and debilitating chemotherapy-induced adverse effect, occurring in 77% of patients receiving doxorubicin-based regimens (Gadisa et al., 2020). The risk of mucositis is further exacerbated when cyclophosphamide and taxane are administered in combination (Menezes et al., 2021).

Pentoxifylline (PTX), a synthetic methylxanthine derivative, has been widely used for the treatment of intermittent claudication. Pentoxifylline has demonstrated significant protective effects against various chemotherapy-induced toxicities. It can protect non-cancerous eukaryotic cells from doxorubicin toxicity, with no protective effect on cancerous cells (Gołuński et al., 2016). Furthermore, a preclinical study suggests that PTX may protect against doxorubicin-induced cardiomyopathy (Zang et al., 2015). Several studies have also highlighted PTX’s potential in reducing mucositis. Clinical observations have reported that PTX can alleviate the severity and duration of mucositis in patients undergoing radiotherapy or chemotherapy (Bianco et al., 1991; Meirovitz et al., 2021). Additionally, PTX exhibits anti-inflammatory and antioxidative properties, which are likely to contribute to its protective effects (Armagan et al., 2015; El Magdoub et al., 2020; Pavitrakar et al., 2022).

Preclinical studies hypothesized that early administration of PTX can provide more comprehensive protective benefits and can delay the onset of neuropathic pain in rats (Kim et al., 2016). It also have a potential effect in reducing post-surgical pain (Dewidar et al., 2025). Recent evidence regarding the use of PTX for the prevention of PIPN is primarily derived from small-scale pilot clinical studies, which have produced conflicting results (Kidwani et al., 2025; Saleh et al., 2024). While some of these studies report promising neuroprotective effects of PTX (Kidwani et al., 2025), others have not demonstrated statistically significant benefits (Saleh et al., 2024). Moreover, previous studies have not explored PTX’s possible role in preventing other common and clinically significant toxicities associated with the AC/T regimen, such as mucositis and cardiotoxicity. These complications are particularly relevant in patients receiving doxorubicin-based chemotherapy and can substantially impact treatment adherence and quality of life.

This study aimed to evaluate the effect of PTX in reducing incidence and severity of peripheral neuropathy, mucositis, and cardiotoxicity in breast cancer patients receiving AC/T regimens of neoadjuvant chemotherapy. A systematic evaluation of PTX’s safety profile and its impact on the overall toxicity burden of the AC/T regimen was also conducted.

## 2 Methods

### 2.1 Study design and settings

This was an open-label randomized trial approved by the ethical committee of the Faculty of Pharmacy, Mansoura University (2023-147). The protocol was registered at https://clinicaltrials.gov/study/NCT06186700 (NCT06186700). The study was conducted according to the Declaration of Helsinki. All patients provided their informed consent before enrollment.

### 2.2 Patients

From December 2023 to August 2024, patients at the Oncology Center of Mansoura University were assessed for eligibility. The study included adults (>18 years) with histologically confirmed primary invasive breast cancer who were scheduled to receive neoadjuvant chemotherapy. Eligible patients had adequate hepatic functions (i.e., aspartate aminotransferase levels not exceeding 2.5 times the upper normal limit, and serum bilirubin levels not higher than 1.5 times the upper normal limit), renal functions (i.e., serum creatinine levels up to 1.4 mg%), and bone marrow functions (i.e., a platelet count exceeding 100 × 10^9^/L and an absolute neutrophil count greater than 1.5 × 10^9^/L). The exclusion criteria were the use of phosphodiesterase inhibitors before enrollment, allergy to PTX, those currently on antiplatelets, patients with history of hemorrhagic events, or those with an active peptic ulcer.

### 2.3 Randomization and study interventions

**FIGURE 1:**
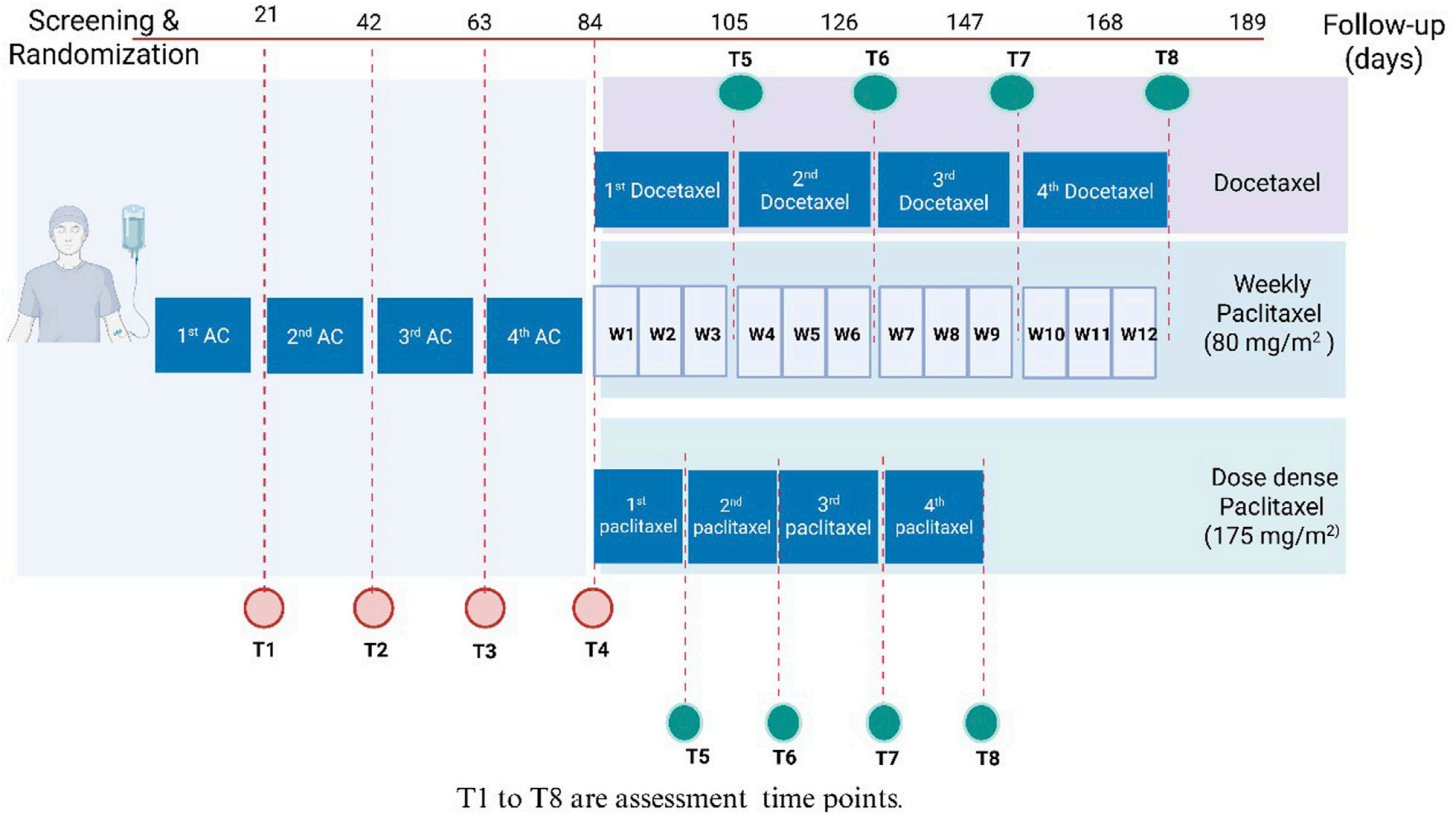
Study design. AC, Adriamycin (doxorubicin) and cyclophosphamide; DD, dose dense; T, time; W, week.

Patients were randomized in (1:1 ratio) following simple randomization procedures, using a computerized random sequence generator, either to the PTX group or the control group. Eligible participants were assigned sequentially based on this list. Patients in the PTX group received 400 mg PTX tablets three times daily (Meirovitz et al., 2021), in addition to the conventional neoadjuvant therapy protocol. Patients in the control group received the conventional neoadjuvant therapy regimen alone. This chemotherapy protocol comprises of four cycles of intravenous doxorubicin (60 mg/m^2^), and IV cyclophosphamide (600 mg/m^2^) *per* cycle, followed by paclitaxel or docetaxel. Paclitaxel is administered either weekly at 80 mg/m^2^ or biweekly in a dose-dense regimen at 175 mg/m^2^. Docetaxel was administered as four cycles with 21 days in between. Patients were interviewed before the administration of each cycle to assess the occurrence of various side effects resulting from the previous cycle. Figure 1 depicts the study design.

### 2.4 Study endpoints

#### 2.4.1 Efficacy outcomes

##### 2.4.1.1 Primary outcome

The primary outcome was the incidence of grade 2 or higher peripheral sensory neuropathy induced by chemotherapy, assessed in both groups throughout the chemotherapy cycles, by the National Cancer Institute-Common Terminology Criteria for Adverse Events (NCI-CTCAE) version 5. Asymptomatic patients were classified as grade 1, those with moderate symptoms limiting instrumental activities of daily living were classified as grade 2, severe symptoms that hindered daily self-care were classified as grade 3, and those with symptoms that posed a potential threat to life were classified as grade 4 (National Cancer Institute, 2017).

##### 2.4.1.2 Secondary outcomes

###### 2.4.1.2.1 The effects on mucositis and cardiotoxicity

The incidence of grade 2 or higher mucositis was assessed throughout the chemotherapy treatment regimen. Based on NCI-CTCAE, mucositis was defined as ulceration or inflammation of the oral mucosa. Patients with mild symptoms or asymptomatic and do not need intervention were classified as grade 1, grade 2 stands for moderate pain or ulcer that did not affect oral intake and only required diet modification, grade 3 included patients with severe symptoms which interfered with oral intake, and grade 4 was for life threatening consequences which indicate urgent intervention. If death occurred, the patient had grade 5 (National Cancer Institute, 2017).

The preventive effect of PTX on cardiomyopathy was evaluated by comparing the ejection fraction of the groups at the end of doxorubicin-containing cycles. Clinically significant cardiotoxicity was defined as a decline in left ventricular ejection fraction (LVEF) ≥10% from baseline to a value < 50% (Kondo et al., 2023; Qiu et al., 2023).

###### 2.4.1.2.2 Quality of life

Validated Arabic-version patient-reported outcome instruments were used to complement clinical evaluation of both peripheral neuropathy and mucositis. Chemotherapy-induced peripheral neuropathy symptoms were assessed using the Functional Assessment of Cancer Therapy–Neurotoxicity (FACT-COG-Ntx) questionnaire (Calhoun et al., 2003) which is known for its validity and reliability in measuring the impact of peripheral neuropathy on patients quality of life (Cheng et al., 2020). The subscale comprises 11 items that assess sensory, motor, and auditory symptoms. Each item is rated on a Likert scale from zero (“not at all”) to four (“very much”). Negatively worded items had reverse scores before calculating the total. By summation of the scores, multiplying by 11, then dividing by the total number of items answered. The Ntx subscale yields a score between 0 and 44, with higher scores indicating better quality of life (Haroun et al., 2023; Kidwani et al., 2025; Mosalam et al., 2020).

The oral mucositis–related quality of life was evaluated using the Oral Health Impact Profile–14 (OHIP-14) which is a 14-question questionnaire that measures the impact of oral health conditions on a person’s quality of life. It assesses the occurrence of negative impacts across seven domains of oral health: functional limitation, physical pain, physical disability, psychological discomfort, psychological disability, social disability, and handicap. Participants respond on a Likert scale indicating frequency. The total score ranges from 0 to 56 and reflects the severity of the perceived oral health impact. Higher scores indicates worse oral health problems (Slade, 1997).

#### 2.4.2 Safety outcomes

##### 2.4.2.1 Effect of PTX on chemotherapy-induced hematological toxicities

The incidence of grade 2 or higher anemia, neutropenia, thrombocytopenia, and febrile neutropenia was assessed by NCI-CTCAE version 5.0 (National Cancer Institute, 2017).

##### 2.4.2.2 Adverse effects of PTX

For hepatic and renal functions, the incidence of elevated alanine aminotransferase (ALT), aspartate aminotransferase (AST), and bilirubin levels was assessed by NCI-CTCAE version 5.0 (National Cancer Institute, 2017). Besides, the incidence of each of the following adverse effects: gastrointestinal symptoms (nausea, vomiting, abdominal discomfort, bloating, diarrhea, constipation), dizziness, and headache was assessed by NCI-CTCAE version 5.0 (National Cancer Institute, 2017).

### 2.5 Patient follow-up and assessment

Patients were interviewed in person before each chemotherapy cycle to check the occurrence of adverse effects and laboratory tests were performed during every cycle of the AC regimen. For those receiving docetaxel or dose-dense paclitaxel, assessments were conducted at each corresponding cycle. In patients treated with weekly paclitaxel, evaluations were carried out every 3 weeks, resulting in a total of eight assessments per patient (Figure 1).

To monitor adherence, a combined approach of routine patient contact between chemotherapy cycles and the collection of empty medication strips to objectively verify compliance and enhance the research integrity. Patients were routinely contacted between cycles to confirm compliance with the prescribed regimen. They were asked to return empty medication strips before receiving the next cycle’s supply. Participants who consumed less than 90% of the scheduled medication were classified as non-adherent and were excluded from the final analysis.

Adverse effects were documented based on clinical assessments, laboratory test results, and a review of patients’ medical records.

### 2.6 Sample size calculation

The sample size was calculated using G*Power (software version 3.1.9.4 Universität Dusseldorf, Germany) and based on a previous study in which the incidence of grade 2 or higher peripheral neuropathy in the PTX group was 28.57% and 64.8% in the control group (Kidwani et al., 2025), using two-sided tests, a power of 0.95, and an alpha error of 0.05. The required sample size was 47 per group, and the total sample size was increased to 110 patients to account for a 15% dropout rate.

### 2.7 Statistical analysis

Data analysis was conducted using Jamovi statistical software, while GraphPad Prism was used for generating graphs. Numerical continuous variables were reported as mean ± SD or median interquartile range (IQR) for non-parametric data following normality assessment using the Shapiro-Wilk test. Categorical data were expressed in terms of frequency and percentage. Data from two groups were compared using an unpaired t-test for normally distributed numerical variables, while the Mann-Whitney U test was employed for numerical data that did not follow a normal distribution. A chi-square test was used for the analysis of categorical data. Absolute risk reduction, number needed to treat, and relative risk of developing neuropathy and mucositis were calculated. Subgroup analysis based on the taxane regimen (weekly paclitaxel, dose-dense paclitaxel, and docetaxel) was performed. The Kaplan-Meier method was used to study the time to incidence of neuropathy, and the log-rank test was used for the significance level. Using a two-sided test, the significance level was set at a P-value of less than 0.05. For variables with less than 10% of missing data, a complete case analysis was carried out, excluding records with missing values. No adjustments were made for multiple comparisons.

## 3 Results

### 3.1 Baseline patient characteristics

**FIGURE 2:**
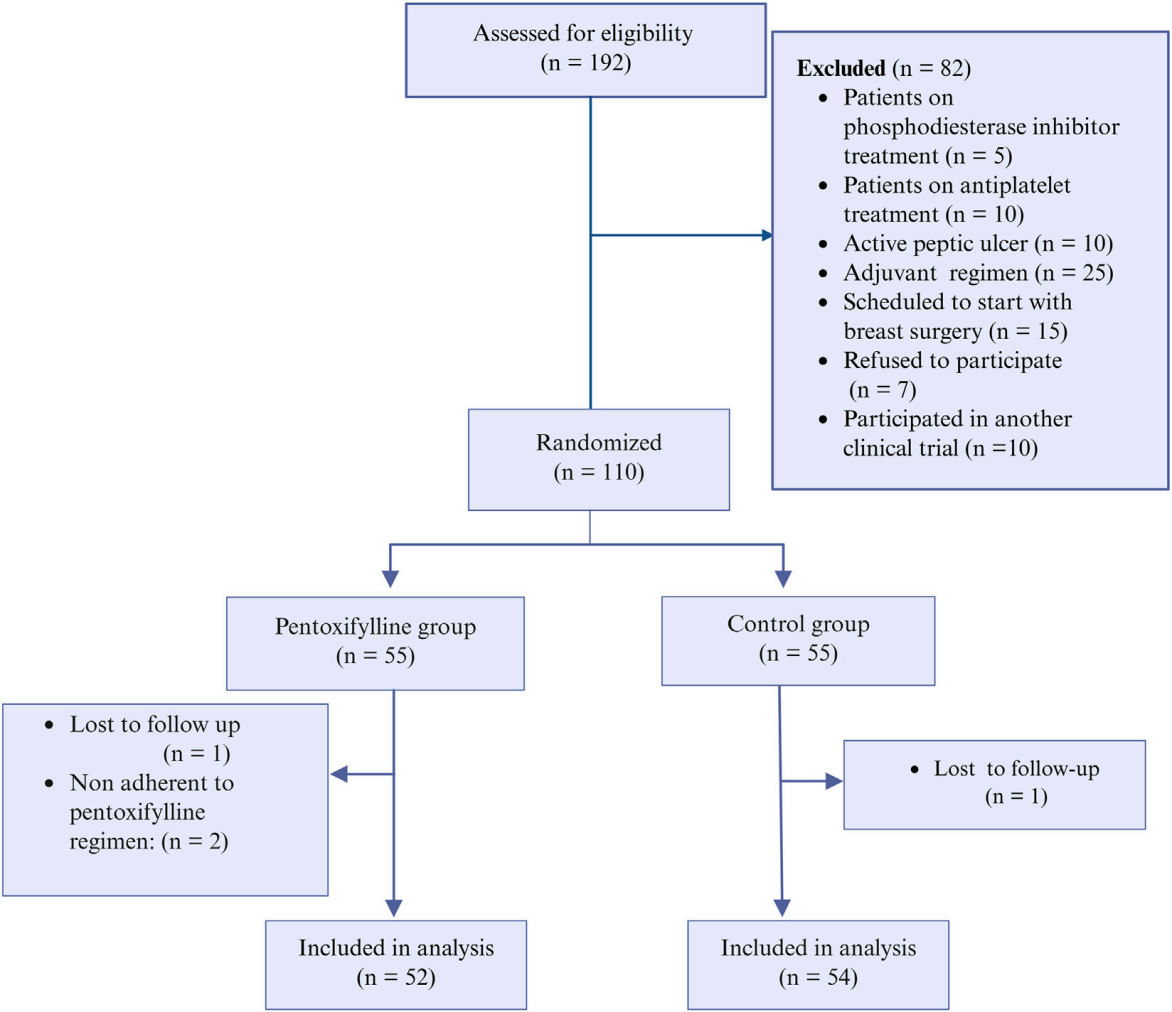
CONSORT flow chart.

Among the screened patients, 110 were randomized into either the PTX group or the control group, resulting in 55 patients per group. After the follow-up, 52 patients in the PTX group and 54 in the control group were included in the final analysis (Figure 2). There were no significant differences in baseline patient characteristics between the two groups. The collected demographic and clinical data included age, family history of breast cancer in first-degree relatives, prior use of oral contraceptives, body surface area, type of taxane regimen received, and the presence of comorbid conditions. Tumor-related characteristics, such as tumor size, histological grade, clinical stage, and both histological and molecular subtypes, were also comparable across groups. Most participants were diagnosed with grade 2 invasive ductal carcinoma, with most tumors classified as the luminal B molecular subtype. Table 1 provides a summary of these baseline characteristics.

**TABLE 1:** Baseline patient characteristics.

Characteristics	PTX (N = 52)	Control (N = 54)	Test statistic
Age (year), mean ± SD	48.2 ± 10.6	51.2 ± 9.99	0.14^a^
Family history of breast cancer	14 (26.9%)	17 (31.5%)	0.61^b^
Contraceptives use	10 (19.2%)	9 (16.7%)	0.73^b^
Type of taxane use			
Docetaxel	11 (21.2%)	7 (13.0%)	0.26^b^
Paclitaxel	41 (78.8%)	47 (87.0%)	
Weekly paclitaxel	15 (28.8%)	21 (38.9%)	0.28^b^
BSA (m^2^), mean ± SD	1.92 ± 0.15	1.96 ± 0.21	0.18^a^
Comorbidity	19 (36.5%)	26 (48.1%)	0.23^b^
Hypertension	11 (21.2%)	21 (38.9%)	0.05^b^
Others	4 (7.7%)	7 (13.0%)	0.37^b^
Tumor side			
Left	21 (40.4%)	24 (44.4%)	0.31^b^
Right	31 (59.6%)	28 (51.9%)	
Bilateral	0 (0.0%)	2 (3.7%)	
Tumor size (mm), median (IQR)	27.3 (22.0–36.9)	29.5 (23.0–41.6)	0.34^c^
Histological subtype			
Invasive duct carcinoma	51 (98.1%)	49 (90.7%)	0.10^b^
Invasive lobular carcinoma	1 (1.9%)	5 (9.3%)	
Tumor grade			
Grade 2	46 (88.5%)	40 (74.1%)	0.06^b^
Grade 3	6 (11.5%)	14 (25.9%)	
Stage			
Stage 1	1 (1.9%)	4 (7.4%)	0.78^b^
Stage 2a	9 (17.3%)	10 (18.5%)	
Stage 2b	20 (38.5%)	17 (31.5%)	
Stage 3a	13 (25.0%)	14 (25.9%)	
Stage 3b	7 (13.5%)	8 (14.8%)	
Stage 3c	2 (3.8%)	1 (1.9%)	
ER: positive	40 (76.9%)	45 (83.3%)	0.41^b^
PR: positive	38 (73.1%)	46 (85.2%)	0.12^b^
HER2: positive	19 (36.5%)	18 (33.3%)	0.73^b^
Ki67 (%) median (IQR)	30.0 (25.0–42.9)	37.5 (25.0–50.4)	0.15^c^
Molecular subtype			
Her2-enriched	5 (9.6%)	4 (7.4%)	0.45^b^
Luminal A	4 (7.7%)	5 (9.3%)	
Luminal B	37 (71.2%)	43 (79.6%)	
Triple negative	6 (11.5%)	2 (3.7%)	
Ejection fraction, median (IQR)	67 (66–68)	67 (65.3–68)	o.44^c^

Results are presented as n (%) or otherwise specified.

PTX, pentoxifylline; SD, standard deviation; BSA, body surface area; Ki67, a proliferation marker; HER2, human epidermal growth factor receptor 2; PR, progesterone receptor; ER, estrogen receptor; IQR, inter quartile range; N, number of non-missing values.

^a^ Unpaired t-test.

^b^ Pearson chi-square test.

^c^ Mann-Whitney U test.

### 3.2 Efficacy outcomes

#### 3.2.1 Primary outcome

**FIGURE 3:**
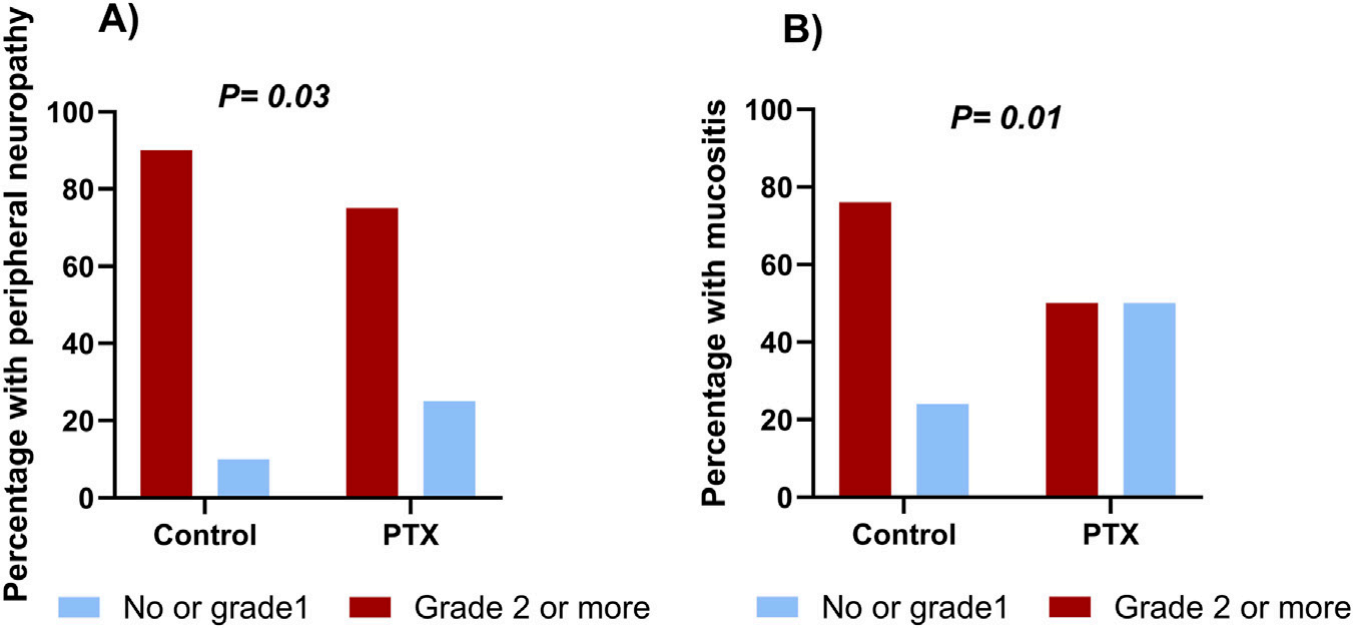
Cumulative incidence of peripheral sensory neuropathy **(A)** and mucositis **(B)** in the pentoxifylline (PTX) and control groups throughout the whole regimen.

As illustrated in Figure 3A, the incidence of grade 2 or higher peripheral neuropathy throughout the whole chemotherapy regimen was significantly lower in the PTX group compared to the control group (39 (75%) vs*.* 49 (90.7%), *P =* 0.03). Throughout the whole treatment regimen, the absolute risk reduction of neuropathy was 15.7% (95% CI: 29.8%–1.7%), corresponding to a number needed to treat (NNT) of 6. The relative risk was 0.83 (95% CI: 0.69–0.99).

Neuropathy incidence was minimal during the AC regimen, without significant difference between the two groups (*P =* 0.18). During the taxane phase, grade 2 or higher neuropathy incidence sharply increased in both arms; however, it was significantly less severe in the PTX group (*P =* 0.03, Table 2). Details regarding the incidence of different grades are present in Supplementary Table S1. Subgroup analysis based on the taxane regimen (weekly paclitaxel, dose-dense paclitaxel, and docetaxel) showed that the PTX-treated patients exhibited numerically lower incidences of grade 2 or higher neuropathy compared to controls, but these differences did not reach statistical significance (*P >* 0.05, Supplementary Table S2).

**TABLE 2:** Primary and secondary endpoints.

Endpoints	PTX (N = 52)	Control (N = 54)	P-value
Neuropathy (AC cycles)			
Grade 2 or higher	1 (1.9%)	4 (7.4%)	P = 0.18^a^
Grade 1	51 (98.1%)	66.0 50 (92.6%)	
Neuropathy (Taxane cycles)			
Grade 2 or higher	39 (75.0%)	49 (90.7%)	P = 0.03^a^
Grade 1	13 (25.0%)	5 (9.3%)	
Mucositis (AC cycles)			
Grade 2 or higher	20 (38.5%)	34 (63.0%)	P = 0.01^a^
Grade 1	32 (61.5%)	20 (37.0%)	
Mucositis (Taxane cycles)			
Grade 2 or higher	7 (13.5%)	16 (29.6%)	P = 0.04^a^
Grade 1	45 (86.5%)	38 (70.4%)	
Ejection Fractions (%), median (IQR)	66.5 (66–68)	66.0 (65–67)	0.33^b^

^a^ Pearson Chi-square test. PTX, pentoxifylline; AC, adriamycin and cyclophosphamide.

^b^ Mann-Whitney U test.

**FIGURE 4:**
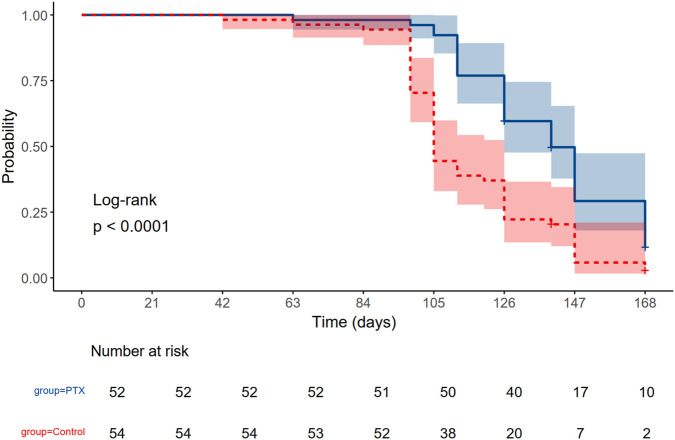
Kaplan Meier estimates of developing grade 2 or higher peripheral neuropathy in the Pentoxifylline (PTX) and Control arm throughout the whole regimen. The *P-value* of log-rank *<*0.0001.

As shown in Figure 4, the probability of remaining free from grade 2 or higher peripheral neuropathy was consistently greater in the PTX group compared to the control group throughout the treatment period especially during the taxane cycles. The log-rank test demonstrated a statistically significant difference between the two groups (P < 0.0001). By day 105, 50% of the control group had developed grade 2 or higher neuropathy. In contrast, 50% of the PTX group did not reach the event until day 140.

#### 3.2.2 Secondary outcome

Neurotoxicity-related quality of life, assessed using the FACT-GOG-Ntx scale, was compared between the PTX and control groups before and after neoadjuvant chemotherapy regimen. At baseline, scores were not significantly different between groups (*P* = 0.06), indicating similar starting points. However, by completing the chemotherapy protocol, the PTX group demonstrated significantly better neurotoxicity-related quality of life compared to controls, *P <* 0.001 (Supplementary Figure S1).

There was a statistically significant difference in mucositis incidence between the study groups throughout the whole chemotherapy regimen, as appeared in Figure 3B (26 (50.0%) in the PTX group vs*.* 41 (75.9%) in the control group, *P =* 0.01). Through the whole treatment regimen, the absolute risk reduction of mucositis was 25.9% (95% CI: 43.7%–8.2%), corresponding to a number needed to treat (NNT) of 4. The relative risk was 0.66 (95% CI: 0.48–0.90).

The PTX group showed a significantly reduced incidence of grade 2 or higher mucositis compared to that reported in the control group in both phases of chemotherapy, as depicted in Table 2; *P =* 0.01, 0.04 during AC and taxane cycles, respectively.

Oral health–related quality of life, measured by the OHIP-14 scale, was comparable between groups at baseline (*P =* 0.15). However, by the end of treatment, patients in the PTX group reported significantly better oral health quality of life (*P <* 0.01, Supplementary Table S3).

After completing the neoadjuvant treatment with the AC regimen, the ejection fraction was 66.5% in the PTX group and 66.0% in the control group (*P =* 0.33). No participants experienced a decline in LVEF meeting the predefined threshold for clinically significant cardiotoxicity.

### 3.3 Safety and adverse effects

#### 3.3.1 Effect of PTX on chemotherapy-induced hematological toxicities

Grade 2 or higher anemia was developed in 11.5% of the PTX group, versus 24.1% of the control group; however, the difference was not significant (*P =* 0.09). Similarly, neutropenia and febrile neutropenia incidences were similar between the study groups (*P =* 0.73, 0.56, respectively*,* Table 3).

**TABLE 3:** The effect of PTX on chemotherapy-induced hematological toxicities.

Hematological toxicities	PTX group N = 52	Control group N = 54	*P-value*
Anemia: Grade 2 or higher	6 (11.5%)	13 (24.1%)	0.09^a^
Neutropenia: Grade 2 or higher	19 (36.5%)	18 (33.3%)	0.73^a^
Febrile neutropenia: Grade 2 or higher	12 (23.1%)	10 (18.5%)	0.56^a^

PTX: pentoxifylline.

^a^ Pearson Chi-square test.

#### 3.3.2 Adverse effects of PTX

Throughout the treatment cycles, two patients developed grade 2 increase in serum creatinine in the PTX group. However, this was not recorded in the control group, and the difference was not significant (*P* = 0.15). There was also no significant increase in levels of all hepatic markers, including bilirubin, AST, and ALT levels (*P* = 0.98, 0.76, 0.63, respectively*,* Table 4).

**TABLE 4:** Effect of PTX on hepatic and renal functions.

Parameter	PTX group N = 52	Control group N = 54	*P-value*
Serum creatinine (mg/dL), (n = 105)			
Not increased	49 (94.2%)	49 (92.5%)	0.15^a^
Grade 1	1 (1.9%)	4 (7.5%)	
Grade 2	2 (3.8%)	0 (0.0%)	
Bilirubin, (n = 105)			
Not increased	48 (92.3%)	49 (92.5%)	0.98^a^
Grade 1	4 (7.7%)	4 (7.5%)	
Alanine Aminotransferase (U/L), (n = 105)			
Not increased	43 (82.7%)	45 (84.9%)	0.76^a^
Grade 1	9 (17.3%)	8 (15.1%)	
Aspartate aminotransferase (U/L), (n = 105)			
Not increased	25 (49.0%)	23 (44.2%)	0.63^a^
Grade 1	26 (51.0%)	29 (55.8%)	

PTX: pentoxifylline. N is the number of non-missing values.

^a^ Pearson chi-square test.

The incidence of grade 2 or higher adverse effects between the PTX and control groups is summarized in Table 5. Gastrointestinal adverse effects, including nausea, vomiting, and abdominal discomfort, were highly prevalent in both groups, with no significant differences observed (*P =* 0.54, 0.37, and 0.37, respectively). Similarly, diarrhea and constipation occur at comparable rates between the groups (*P* = 0.45 and 0.17, respectively).

**TABLE 5:** Incidence of grade 2 or higher adverse effects of pentoxifylline and chemotherapy.

Adverse effects of PTX	PTX (N = 52)	Control (N = 54)	*P value*
Gastrointestinal adverse effects: grade 2 or higher			
Nausea	50 (96.2%)	53 (98.1%)	0.54^a^
Vomiting	48 (92.3%)	52 (96.3%)	0.37^a^
Abdominal discomfort	48 (92.3%)	52 (96.3%)	0.37^a^
Diarrhea	6 (11.5%)	9 (16.7%)	0.45^a^
Constipation	22 (42.3%)	16 (29.6%)	0.17^a^
Bloating	27 (51.9%)	36 (66.7%)	0.12^a^
Headache: Grade 2 or higher	51 (98.1%)	51 (94.4%)	0.33^a^
Flushing: Grade 2 or higher	9 (17.3%)	5 (9.3%)	0.22^a^

PTX: pentoxifylline. N is the number of non-missing values.

^a^ Pearson chi-square test.

Although bloating was more frequently reported in the control group (66.7% vs. 51.9% in PTX), the difference was not statistically significant (*P =* 0.12). Headache incidence was almost equal in both groups (98.1% in PTX vs. 94.4% in control, *P =* 0.33), while flushing was slightly more common in the PTX group (17.3% vs. 9.3% in control, *P =* 0.22), though this difference did not reach statistical significance (Table 5). No treatment withdrawals or dose-modifications were required due to adverse reactions attributed specifically to PTX.

## 4 Discussion

This study aimed to evaluate the use of PTX to protect from chemotherapy-induced peripheral neuropathy throughout the regimen of AC/taxane in neoadjuvant breast cancer patients. The primary endpoint was the difference in the incidence of grade 2 or higher peripheral neuropathy, assessed based on the NCBI-CTCAE (Li et al., 2022; National Cancer Institute, 2017) that was widely used in previous clinical trials (Bakry et al., 2023; Haroun et al., 2023; Li et al., 2022; Werida et al., 2022).

The incidence of peripheral neuropathy was minimal in the AC cycles compared to the taxane regimen, and it was comparable for the two study groups (*P =* 0.18), which is consistent with the previous studies (Gadisa et al., 2020). With the initiation of the taxane regimen, the incidence of neuropathy increased in both groups with higher percentage in the control group (75% in PTX vs. 90.7% in control group; *P =* 0.03). However, across all taxane regimen subgroups, the PTX-treated patients exhibited numerically lower rates of grade ≥2 neuropathy compared to controls. While these differences did not reach statistical significance (*P >* 0.05), possibly due to limited sample sizes within each subgroup, there was a consistent trend suggesting a potential protective effect of PTX, particularly with dose-dense paclitaxel and docetaxel. There was also a significantly longer time to develop symptoms of neuropathy in the PTX arm compared to that recorded in the control arm (*P <* 0.0001), suggesting that PTX may exert a protective effect against chemotherapy-induced peripheral neuropathy. In search of an underlying mechanism, the possible reasons have been investigated in other preclinical and clinical studies. PTX alleviated paclitaxel-induced mechanical allodynia in the rat model by reducing inflammatory cytokines such as TNF-α and IL-1β in the lumbar dorsal root ganglia (Kim et al., 2016). Similar findings attributed to another clinical trial that assessed the preventive capacity of PTX against the development of peripheral neuropathy in patients using a weekly paclitaxel regimen (Kidwani et al., 2025). They found that 28.6% of subjects in the PTX group developed grade 2 or higher peripheral neuropathy compared to 64.9% in the control group (*P =* 0.016). These findings are consistent with previous research investigating the neuroprotective effects of various phosphodiesterase inhibitors. Ibudilast, a phosphodiesterase 4 inhibitor, has been shown to attenuate paclitaxel-induced peripheral neuropathy in mice models by restoring intracellular calcium homeostasis in sensory neurons. This is achieved through the mitigation of paclitaxel-induced disruption of neuronal calcium sensor 1, thereby preserving calcium signaling integrity (Sisignano et al., 2016). Similarly, cilostazol, a phosphodiesterase-3 inhibitor, has demonstrated protective effects in preclinical settings by promoting Schwann cell differentiation via the cAMP/Epac signaling pathway, thereby preventing paclitaxel-induced dedifferentiation and demyelination (Koyanagi et al., 2021). These preclinical findings have been corroborated by clinical evidence, where cilostazol was shown to mitigate paclitaxel-induced neurotoxicity (Haroun et al., 2023).

In contrast, the findings of Saleh et al. (2024) did not align with the results of the present study regarding the incidence of grade 2 or higher peripheral neuropathy, which was reported to be comparable between the groups in their study. This discrepancy may be explained by using a smaller sample size, which was calculated based on the change in TNF alpha rather than the incidence of neuropathy as the primary outcome. Moreover, the full dose of PTX was not administered throughout the treatment period (Saleh et al., 2024).

The current clinical study also highlights the possible effect of PTX in reducing the incidence of grade 2 or higher mucositis among patients receiving the AC/T regimen. This protective effect may be attributed to the effect of PTX on the reduction of reactive oxygen species (Armagan et al., 2015; El Magdoub et al., 2020; Pavitrakar et al., 2022), which are known to cause DNA damage and subsequent basal epithelial cell death in the oral mucosa (Saito et al., 2022). In addition to its antioxidant activity, PTX also exhibits anti-inflammatory and mucosa-protective properties (Gruber et al., 2017; Lima et al., 2005; Price et al., 2020) as supported by a recently published study (Wu et al., 2025). Price et al. demonstrated that PTX administration in mice not only inhibited TNFα signaling but also alleviated oral mucositis-induced pain following radiotherapy (Price et al., 2020). Similarly, other preclinical models have shown that PTX significantly reduces the expression of TNFα and IL-1β, thereby promoting the healing of radiotherapy-induced oral ulcers (Gruber et al., 2017). In a hamster model of 5-FU-induced oral mucositis, local subcutaneous injections of PTX markedly decreased the severity of mucositis, as evidenced by reductions in hyperemia, erythema, edema, inflammatory infiltration, and ulceration (Lima et al., 2005).

The findings of the present study align with a previous study on colon cancer patients receiving chemotherapy, which stated that PTX could significantly diminish the incidence of stomatitis in the study patients from 45.5% in the control group to 25% when PTX was administered (Bianco et al., 1991; Meirovitz et al., 2021). Similarly, our findings are in line with those of another clinical trial involving head and neck cancer patients, which demonstrated that a combination of oral PTX and vitamin E significantly reduced both the severity and duration of acute radiotherapy-induced oral mucositis and dysphagia (Sayed et al., 2019). Despite these supporting studies, prior evidence has not been uniformly supportive. A systematic review discouraged the use of systemic PTX for preventing oral mucositis, particularly in patients undergoing stem cell transplantation. However, that recommendation was based largely on studies in hematological malignancies, where the toxicity profiles differ markedly from those in solid tumor chemotherapy. Notably, only one small trial included in that review involving patients with solid tumors (n = 10 patients), limiting the strength of its conclusions. These differences in patient populations, and sample sizes likely account for the discrepancy and underscore the need for further targeted studies in solid tumor patients.

The potential cardioprotective effect of PTX observed in preclinical studies was not replicated in the clinical setting of this study. The ejection fraction post-treatment with doxorubicin was similar between the groups (*P =* 0.33), indicating that the co-administration of PTX with doxorubicin-containing regimens does not offer additional benefits regarding cardiotoxicity, despite preclinical evidence supporting the protective role of PTX against doxorubicin-induced cardiac damage (Elshazly et al., 2016; Gołuński et al., 2016). These preclinical findings were supported by histological assessments showing reduced myocardial fibrosis and apoptosis.

Hematological toxicity is a widely known problem for chemotherapy regimens including doxorubicin, cyclophosphamide, and taxane (Fikremariam Abiye and Abebaye Aragaw, 2023; Simone Yuriko et al., 2021), caused primarily by suppressing bone marrow function or triggering immune-mediated hemolysis. In the present study, the rates of hematological toxicities did not differ significantly between the treatment groups. Although the incidence of anemia was higher in the control group compared to the PTX group (24.1% vs. 11.5%, respectively), this difference did not reach statistical significance (*P =* 0.09). Similarly, the incidences of neutropenia and febrile neutropenia were comparable between groups (*P =* 0.73 and *P =* 0.56, respectively). This means that PTX did not increase the incidence of hematologic toxicity compared to the control group. This finding is consistent with the results of a randomized controlled trial conducted in bone marrow transplant patients, which reported no significant differences in transfusion requirements or other hematologic outcomes between the study groups (Attal et al., 1993). Additional studies have also highlighted the potential hematologic benefits of PTX, particularly its favorable effects on anemia. For example, PTX has shown promise in improving anemia in hemodialysis patients (Mora-Gutierrez et al., 2013) and in individuals with chronic kidney disease (Bolignano et al., 2015).

The findings of this study indicate that PTX does not contribute to renal toxicity when co-administered with chemotherapy. A comparison of patients who experienced a grade 2 increase in serum creatinine revealed a similar incidence between the two groups, with 2 patients (3.8%) in the PTX group and none (0.0%) in the control group (*P =* 0.15). This finding suggests that the administration of PTX did not exacerbate renal dysfunction in the study population. In agreement with this observation (Attal et al., 1993), reported that the addition of PTX did not affect the incidence of renal insufficiency. Furthermore, other studies have demonstrated the potential reno-protective effects of PTX. For instance, PTX has been shown to slow the progression of chronic kidney disease (Christian et al., 2016), stabilize renal function, and reduce proteinuria (Alejandra Muñoz de et al., 2019).

In the present study, the incidence of common adverse effects was comparable between the two study groups. This observation is consistent with previously published research, which has consistently demonstrated that PTX possesses a safety profile similar to that of a placebo. A systematic review of nine placebo-controlled trials involving patients with intermittent claudication reported that the most frequently observed adverse effects of PTX, gastrointestinal symptoms, headache, and dizziness, occurred at rates comparable to those in placebo groups (Salhiyyah et al., 2015). Overall, PTX is considered generally well tolerated, with most reported adverse events being mild in nature (Semmia et al., 2023) and this matches our findings that PTX did not differ from the control group in tolerability. This favorable safety profile supports the clinical utility of PTX, particularly when considering its risk–benefit ratio in mitigating chemotherapy-associated toxicities and its potential therapeutic value in oncology settings (Golunski et al., 2018).

This study is the first to investigate the potential effect of PTX on the various toxicities experienced throughout the entire neoadjuvant chemotherapy protocol in breast cancer patients. Nonetheless, several limitations should be acknowledged. First, the sample size was relatively small, which may limit the generalizability of the findings. Notably, while the study was powered for the primary endpoint of peripheral neuropathy, it may be underpowered to detect differences in the secondary outcomes including cardiotoxicity and hematological effects. Second, the study was conducted with an open-label design which may introduce a risk of performance and outcome detection bias. Additionally, the study lacked allocation concealment, potentially leading to selection bias. Third, this study exclusively examined acute peripheral neuropathy following chemotherapy. Thus, further studies are warranted to evaluate the potential effects of PTX in chronic neuropathy. Cardiac safety was only assessed by ejection fraction, which may miss subclinical toxicity. Hepatic and renal function were monitored using standard labs, though these may not detect subtle or long-term effects. Finally, this study was conducted at a single center, enrolling exclusively non-metastatic breast cancer patients undergoing neoadjuvant chemotherapy; therefore, the findings may only be generalized to similar clinical settings.

## 5 Conclusion

In conclusion, the addition of PTX to neoadjuvant chemotherapy regimens like AC/Taxane shows promise in reducing the incidence of grade 2 or higher chemotherapy-induced peripheral neuropathy and reducing the occurrence of grade 2 or higher mucositis without causing additional side effects in breast cancer patients.

## Data Availability

The raw data supporting the conclusions of this article will be made available by the authors, without undue reservation.
