# Supplementary material for: Assessing the efficacy and safety of pentoxifylline in preventing chemotherapy-induced peripheral neuropathy and mucositis in breast cancer patients
Source: Front Pharmacol. 2025 Oct 14;16:1678161. doi: 10.3389/fphar.2025.1678161 (PMC12558964; doi:10.3389/fphar.2025.1678161)
Supplement: Supplementary file 1 [file DataSheet1.pdf]

Supplementary data

Table S1: Detailed incidence of neuropathy and mucositis grades in the study groups

	PTX (N=52)	Control (N=54)	P-value
Neuropathy (AC cycles)			P=0.18[2]
Grade 1	51(98.1%)	50(92.6%)	
Grade 2	1(1.9%)	4 (7.4%)	
Grade 3	0(0.0%)	0 (0.0%)	
Grade 4	0(0.0%)	0 (0.0%)	
Grade 5	0(0.0%)	0 (0.0%)	
Neuropathy (Taxane cycles)			**P<0.01**[2]
Grade 1	13(25.0%)	5(9.3%)	
Grade 2	37(71.2%)	28 (51.9%)	
Grade 3	2(3.8%)	21 (38.9%)	
Grade 4	0(0.0%)	0 (0.0%)	
Grade 5	0(0.0%)	0 (0.0%)	
Mucositis (AC cycles)			**P=0.04**[2]
Grade 1	32(61.5%)	20(37.0%)	
Grade 2	16 (30.8%)	27 (50.0%)	
Grade 3	4 (7.7%)	7 (13.0%)	
Grade 4	0 (0.0%)	0 (0.0%)	
Grade 5	0 (0.0%)	0 (0.0%)	
Mucositis (Taxane cycles)			**P=0.04**[2]
Grade 1	45(86.5%)	38(70.4%)	
Grade 2	4 (7.7%)	14 (25.9%)	
Grade 3	3 (5.8%)	2 (3.7%)	
Grade 4	0 (0.0%)	0 (0.0%)	
Grade 5	0 (0.0%)	0 (0.0%)	

[2]Pearson Chi-square test. PTX: pentoxifylline.

Table S2. Incidence of peripheral neuropathy subgroup analysis based on taxane regimen

	PTX	Control	P-value
**Weekly paclitaxel**	(N=15)	(N=21)	P=0.81[2]
Grade 2 or higher	14 (93.3%)	20 (95.3%)	
Grade 1	1 (6.7%)	1 (4.8%)	
**Dose dense paclitaxel**	(N=26)	(N=26)	P=0.11[2]
Grade 2 or higher	17 (65.4%)	22 (84.6%)	
Grade 1	9 (34.6%)	4 (15.4%)	
**Docetaxel**	(N=11)	(N=7)	P=0.13[2]
Grade 2 or higher	8 (72.7%)	7 (100%)	
Grade1	3 (27.3%)	0 (0.0%)	

[2]Pearson Chi-square test. PTX: pentoxifylline.

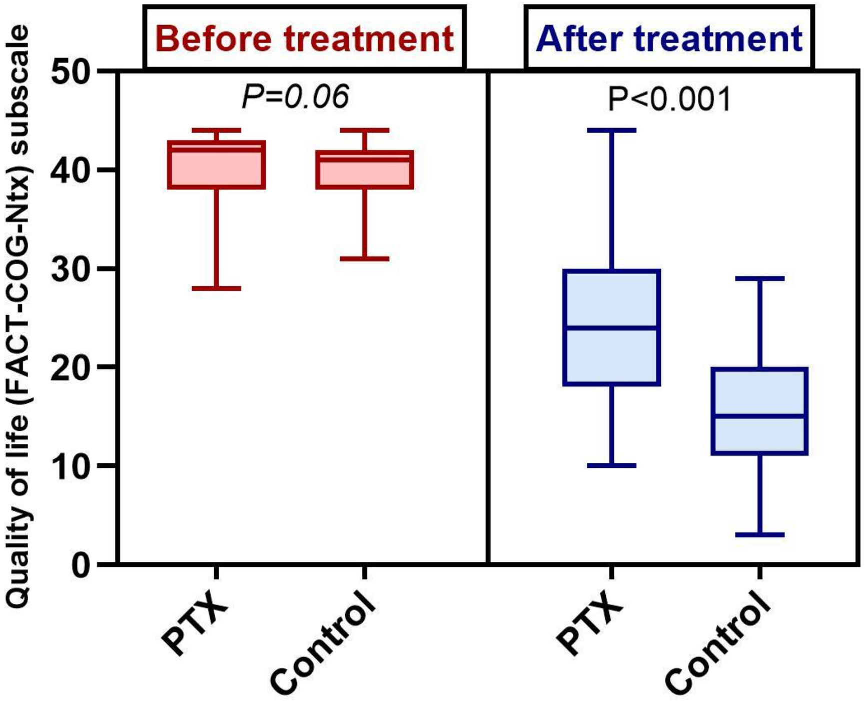

Figure S1. Box plot comparing the quality of life expressed by the Functional Assessment of Cancer Therapy/Gynecologic Oncology Group-Neurotoxicity (FACTGOG- Ntx) in the control and pentoxifylline arms.

Table S3. The oral health quality of life expressed by Oral Health Impact Profile-14(OHIP-14) questionnaire in the pentoxifylline (PTX) and the control arms.

	**PTX (N=52)**	**Control (N=54)**	P-value
**OHIP-14 score before treatment**	0.0 (0.0-0.0)	0 (0.0-0.0)	P=0.15[1]
**OHIP-14 Score after treatment**	10.5 (5.57-16.0)	16.5 (9.25-21.8)	P<0.01[1]

[1]Mann-Whitney U test. PTX: pentoxifylline.
